# Evaluation of a patient-centered communication skills training for nurses (KOMPAT): study protocol of a randomized controlled trial

**DOI:** 10.1186/s12912-023-01660-8

**Published:** 2024-01-02

**Authors:** Anja Lindig, Kendra Mielke, Wiebke Frerichs, Katja Cöllen, Levente Kriston, Martin Härter, Isabelle Scholl

**Affiliations:** 1 Department of Medical Psychology, University Medical Center Hamburg-Eppendorf, Martinistraße 52, 20246 Hamburg, Germany; 2 Center for Health Care Research and Public Health, University Medical Center Hamburg-Eppendorf, Martinistraße 52, 20246 Hamburg, Germany

**Keywords:** Nursing, Communication, Communication skills training, Patient-centeredness, Implementation research, Randomized controlled trial, Mixed methods, Hybrid type 1 effectiveness-implementation design, Train-the-trainer, Self-efficacy

## Abstract

**Background:**

To ensure high quality of nurses’ communication as part of patient-centered care, training of communication skills is essential. Previous studies indicate that communication skills trainings can improve communication skills of nurses and have a positive effect on emotional and psychological burden. However, most show methodological limitations, are not specifically developed for nurses or were developed for oncological setting only.

**Methods:**

This study aims to evaluate the effectiveness of a needs-based communication skills training for nursing professionals and to derive indications for future implementation. A two-armed randomized controlled trial including components from both effectiveness and implementation research will be applied. Additionally, a comprehensive process evaluation will be carried out to derive indications for future implementation. Nurses (*n=*180) of a university medical center in Germany will be randomized to intervention or waitlist-control group. The intervention was developed based on the wishes and needs of nurses, previously assessed via interviews and focus groups. Outcomes to measure effectiveness were selected based on Kirkpatrick’s four levels of training evaluation and will be assessed at baseline, post-training and at 4-weeks follow-up. Primary outcome will be nurses’ self-reported self-efficacy regarding communication skills. Secondary outcomes include nurses’ communication skills assessed via standardized patient assessment, knowledge about patient-centered communication, mental and work-related burden, and participants’ satisfaction with training.

**Discussion:**

To our knowledge, this is the first study systematically evaluating the effectiveness of a patient-centered communication skills training for nursing professionals in Germany. Results will yield insight whether a needs-based intervention can improve nurses’ self-efficacy regarding communication skills and other secondary outcomes.

**Trial registration:**

Clinical trial registration number: NCT05700929, trial register: ClinicalTrials.gov (date of registration: 16 November 2022).

## Introduction

To ensure high quality of nurses’ communication as part of patient-centered care, training of communication skills is essential. Previous studies indicate that communication skills trainings (CST) can improve communication skills of nurses and have a positive effect on emotional and psychological burden. However, most CSTs show methodological limitations, are not specifically developed for nurses or were developed for oncological setting only. This study will contribute to this research gap by systematically evaluating a needs-based CST on patient-centered communication, developed with and for nurses in Germany.

### Background

Central tasks in nurses’ daily work are interaction and communication with patients [1]. Patient-centered communication can improve trusting relationships between nurses and their patients [2], can help to promote positive health outcomes of patients [3], and is crucial for high quality patient-centered nursing practice [4]. Several clinical situations were identified as especially challenging for an adequate patient-centered communication: adequately responding to wishes and needs of patients and their relatives, communicating with seriously ill or dying patients and their relatives, responding to angry and demanding patients and their relatives, and communicating with patients who deny their disease [5, 6]. Handling those situations might be especially challenging if nursing professionals also have to cope with increased workload and reduced workforces. This might hinder addressing needs of patients and delivering patient-centered care appropriately [7, 8].

Several previous studies have indicated the need for a specific training for nursing professionals to enhance communication skills with patients and their relatives [8, 9]. There is evidence that training to enhance communication skills can increase self-efficacy and patient-centered communication skills [10, 11]. Furthermore, a study by Onan et al. [12] found a negative significant correlation between self-assessed communication skills and perceived stress as well as a negative significant correlation between subjectively assessed communication skills and psychological symptoms (i.e. anxiety, depression, negative self-concept, somatization, hostility) of nurses. In a recent Danish study, a large-scale three-day CST was applied to more than 1000 healthcare professionals (HCPs) of different professions and departments [11]. This study found statistically significant higher self-efficacy of HCPs immediately after the training compared to baseline. This effect was strongest for nurses, nurse assistants, physiotherapists and occupational therapists. When evaluating self-efficacy of participants 24 weeks after the training, there was a small but statistically significant decrease in self-efficacy compared to immediate post-training assessment. Yet, self-efficacy scores were still significantly higher compared to baseline assessment. Congruently, an US-American study found that self-efficacy and communication skills of 340 oncological nurses were significantly increased after participating in a CST [10]. A recent systematic review, which included randomized controlled trials (RCT) only, summarized that CSTs for nurses can increase their communicative competencies and can lead to more patient-centered encounters [13]. However, the quality of the included studies was described as modest and evaluated trainings varied largely in duration, structure and applied measures. Additionally, most international studies on evaluating CSTs either focused on oncological settings [10, 13] or did not address nurses exclusively [11].

In Germany, only few CSTs for nurses have been developed and evaluated so far. Haberstroh et al. [14] found that a CST developed for geriatric nurses to improve communication in dementia care increased social competencies of nurses, reduced their psychological burden and increased health-related quality of life of patients with dementia. Berger-Höger et al. [15] developed a specific nurse-led coaching to enhance shared decision-making communication skills for women with breast cancer and evaluated this training in a cluster-RCT. Nurses who were trained as decision coaches reported a better cooperation with physicians and a strengthening of their role as nurses. Another study applied a training for physicians and nurses to deal with bereaved relatives after a sudden death [16]. However, this study lacked a comprehensive evaluation of the training and showed reduced validity due to limitations of the study design.

Since 2017, the Law for Nursing Education in Germany (§ 5 Absatz 1 Satz 1 PflBRefG) defined the acquisition of communication skills as a learning goal. In order to enable nurses from previous education cohorts to acquire equal skills, it is important to offer additional training opportunities. Trainings on communication skills for certified oncology nursing staff are already offered throughout Germany. But quality and quantity of those trainings are heterogeneous [17]. Often, they were not developed based on needs of the target group, missed systematic evaluation with high methodological quality or did not apply effective didactic methods like role plays or video feedback [18]. According to the Consolidated Framework for Implementation Research (CFIR) by Damschroder et al. [19], it is recommended to assess needs of the target group prior to an intervention development. Thus, we conducted a qualitative needs assessment prior to this RCT, which results will be published elsewhere. Participating nurses were asked, which situations in communication with patients they considered particularly challenging and which topics should be addressed in a communication skills training. Analysis of the conducted interviews (*n=*17) with *n=*18 department managers and ward managers and focus groups (*n=*5) with *n=*33 nurses indicated a considerable need for CSTs for nursing professionals. The interviewed nurses reported that difficulties in communication often arise due to inadequate organizational procedures or due to very demanding or aggressive patients and their relatives. Additionally, nurses often find it difficult to manage their own emotions appropriately in such situations. Therefore, nurses’ wishes regarding the CST included information on communication techniques and their adaptation to situations with patients and relatives, especially for de-escalation. Similar training content was requested by department managers. They wished for trainings including dealing with aggressive or demanding patients and their relatives, communication with seriously ill or dying patients and communication under stress and time pressure [20]. Perceived barriers for participating in a CST included negative attitudes towards the importance of patient-centered communication in daily work, limited time resources and insufficient promotion of the training. However, incentives (e.g. attendance certificates, education credits) or transferability of trainings content to daily work were described as facilitators for participation.

To sum up, there is a high need for CSTs developed with and for nursing professionals in Germany to enhance their patient-centered communication skills including a systematic evaluation of high methodological quality. Therefore, based on the results of the qualitative needs assessment and review of the current literature, we developed a CST for nursing professionals in Germany.

## The study

### Aims

The primary aim of this study is to investigate the effectiveness of a newly developed, needs-based patient-centered CST for nurses in Germany based on Kirkpatrick’s four levels of training evaluation [21]. The subsequent research questions are related to the four levels of the Kirkpatrick model:

1. Level: Reaction – How do participants’ feel about the CST (e.g. satisfaction, acceptability)?
2. Level: Learning – What is the impact of the CST on participants’ self-efficacy regarding communication with patients, knowledge about patient-centered communication, communication skills and attitudes towards medical communication?
3. Level: Behavior – What is the impact of the CST on participants’ communication behavior with patients?
4. Level: Results – What is the impact of the CST on participants’ work-related mental burden?

Secondary aim of this study is to derive indications for future implementation of the above-mentioned needs-based patient-centered CST for nurses in Germany. This aim is specified by the following research questions:

1. Which factors may influence the roll-out of the CST?
2. Which indications can be derived for future implementation of the CST?

### Design

#### Study design

This study uses a hybrid type 1 effectiveness-implementation design [22]. The hybrid type 1 effectiveness-implementation design is a combined design including components from both effectiveness and implementation research. By applying a 2-armed RCT with an intervention group (IG) and a waitlist-control group (CG), the effectiveness of a newly developed CST for nursing professionals will be evaluated [23]. To collect indications for future implementation of the training program in routine clinical practice, we will perform a comprehensive process evaluation by collecting qualitative and quantitative data.

**Fig. 1:**
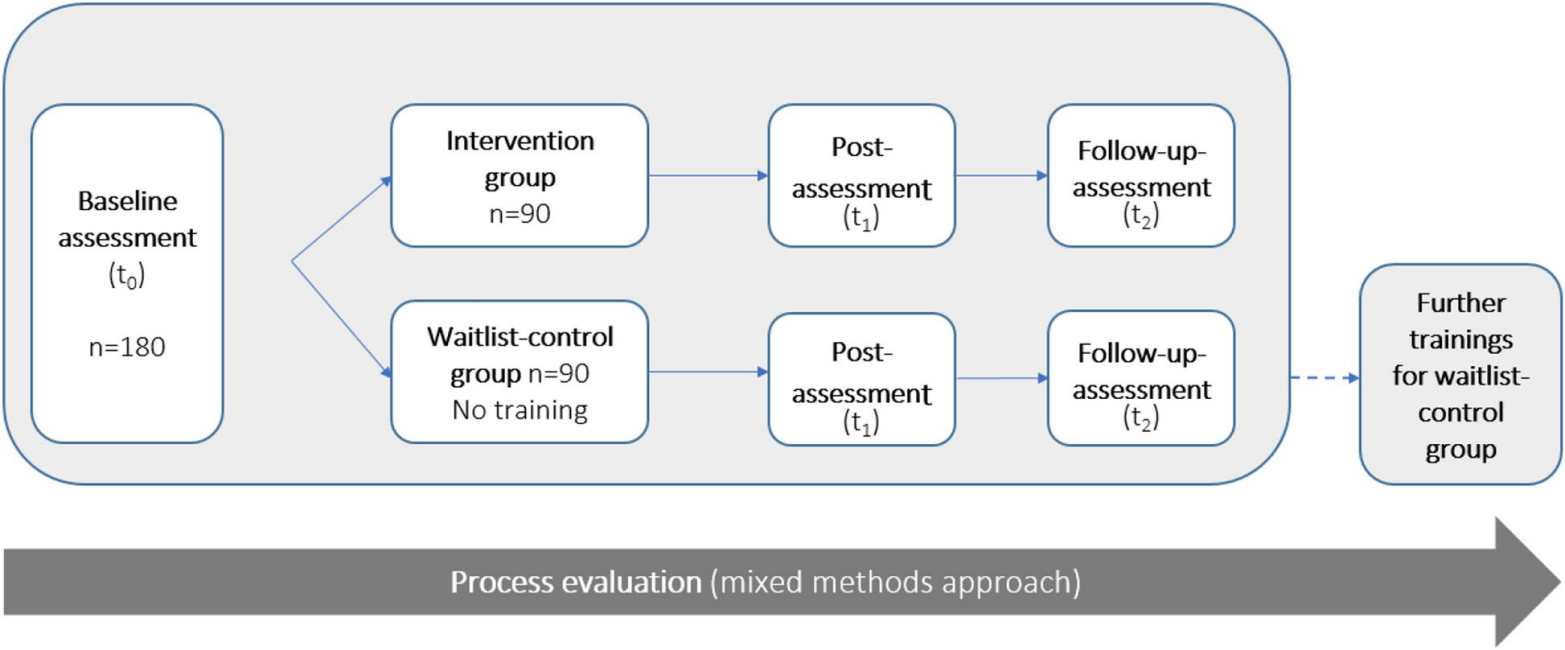
Study design of the hybrid type 1 effectiveness-implementation design, using a randomized controlled trial

For an overview on the study design, see Fig. 1.

#### Setting and participants

The study will be conducted at the University Medical Center Hamburg-Eppendorf (UKE), Germany, an academic medical center in Northern Germany. Participants will be certified nurses working at the UKE. In order to participate in the study, participants are required to have completed an educational program as a nurse (either in the field of regular professional nursing, pediatric nursing or geriatric nursing). Moreover, they need to communicate with patients or relatives during their daily work. Eligible participants must be older than 18 years and speak German sufficiently in order to fill out the surveys and participate in the training. Nursing professionals who have little or no direct contact with patients in their daily work (e.g. operating room nurses) are not eligible for this study. In addition, nursing professionals who have not yet completed their professional education in the above-named fields will be excluded from the study.

#### Intervention

The intervention, a 6-hour face-to-face CST, was developed based on theoretical and empirical findings of a literature search on CSTs for nursing professionals and results of the qualitative needs assessment conducted prior to this study (see background section). We applied an adapted version of the Calgary-Cambridge guide (C-CG) [24] as theoretical framework for the training. The C-CG provides a comprehensive approach for effective teaching and learning of clinical communication skills and has been used in other CSTs for HCPs [11, 25]. The authors defined specific skills which help HCPs to enhance communication with patients and therefore to foster patient-centered communication. Those skills will be taught by using various didactic techniques adapted from other CSTs skills trainings [10, 11, 14], i.e. video sequences for case scenarios, role-play exercises and group discussions. Previous studies have demonstrated that timely feedback is highly valued by participants [26]. Thus, during role-play exercises, participants will receive structured, integrated feedback from participants and the trainers [27].

**Fig. 2:**
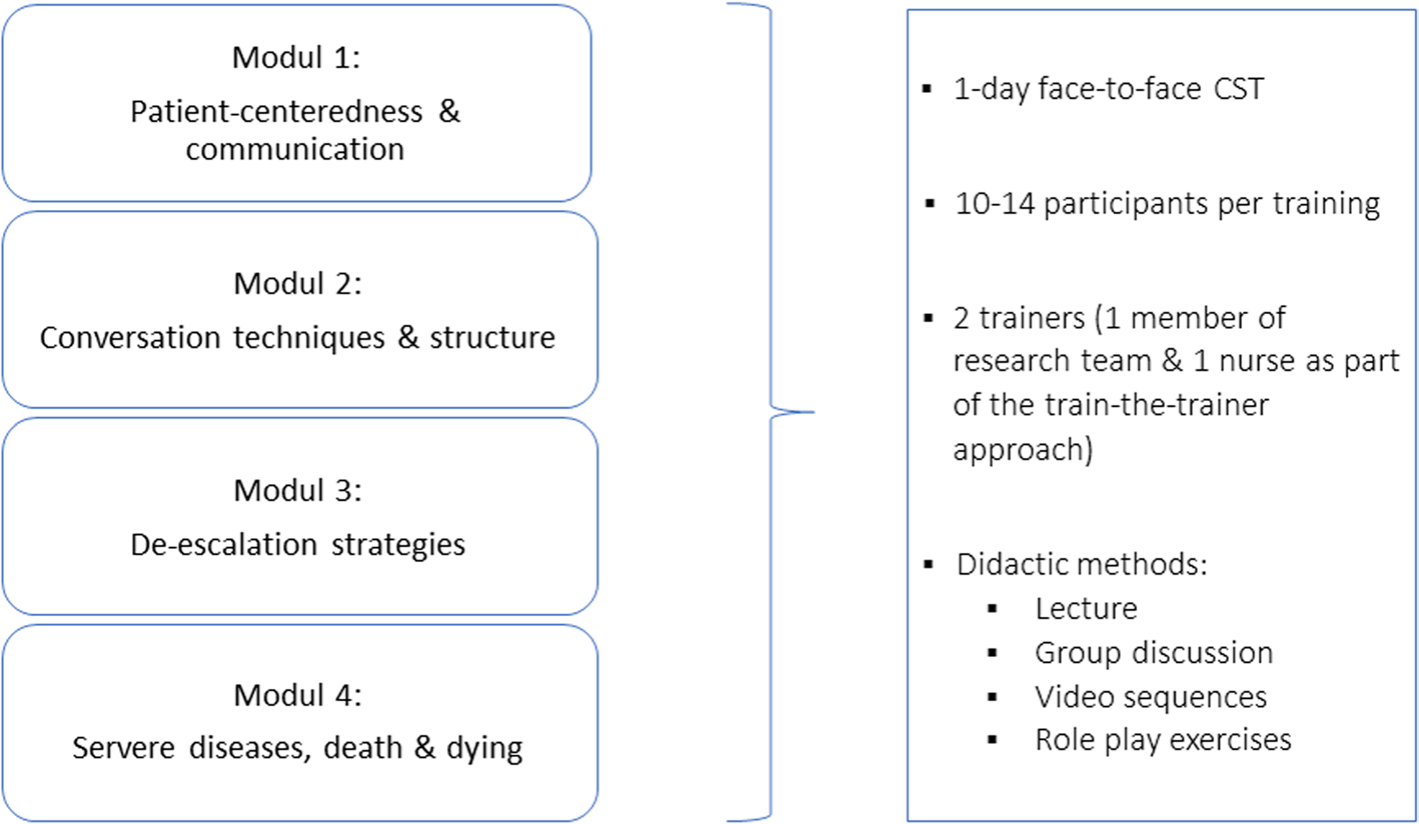
Content, didactic methods and general conditions of the training to enhance nurses’ patient-centered communication skills

The training consists of four modules. In module 1, basic information regarding patient-centeredness and communication will be provided. In module 2, participating nurses will receive information on general conversation techniques (e.g. non-verbal communication). In module 3, participants will learn how to response to patients in challenging situations (e.g. de-escalation in communication). Module 4 will focus on communication with seriously ill or dying patients and their relatives (see Fig. 2). All participants will receive a manual about the content of the training.

The training will be piloted with 8 to 12 nurses and other experts in the field of training development and finalized afterwards. It will be carried out in groups of 10 to 14 participants each and conducted by two trainers. The number of participants per training group will be planed slightly higher with 14 participants, as experience has shown that some participants cancel at short notice. One of the trainers will be a member of the research team with comprehensive experience in CSTs, as well as experience in communicating with patients. The second trainer will be a nursing professional, recently working at the UKE. We will use a train-the-trainer approach, meaning nurses will be trained in order to be able to conduct the communication skills training together with the research team. The train-the-trainer approach was found to enhance implementation success and sustainability of CST interventions [13].

#### Sample size

Based on results of previous studies on effects of CSTs on self-efficacy of nurses, we aim to identify a moderate effect of the intervention (Cohen’s d of 0.50) on the primary outcome [28, 29]. To identify this effect in a two-sided test with an alpha error probability of 0.05 and a power of 0.80, data from a total sample of 128 participants (64 participants per group) are needed. In order to be able to account for missing data due to participant dropout, for potential differences between treatment groups at baseline, and for clustered data structures, we aim to recruit a total of 180 participants (90 participants in each group).

#### Recruitment and randomization

Nurses will be recruited continuously starting in November 2022 until spring 2024 within their departments. Therefore, we contact heads of relevant departments at the UKE by e-mail and ask them to forward the study information to all eligible staff in the respective department. Prior to study participation, eligible nurses will be informed that there is an equal chance to be assigned to the IG or CG. After giving informed written consent, they will be asked to complete the baseline assessment (t0). Afterwards, participants will be randomized in a 1:1 ratio to either IG or CG. For this purpose, stratified randomization will be applied [30]. Each department of the UKE will be defined as a stratum. A member of the research team, who will not be involved in recruitment of eligible nurses, will allocate participating nurses within each stratum to either IG or CG by using permuted blocks. Participating nurses of the CG are offered to participate in the training after data collection has been completed.

### Outcomes and measures

We will carry out a mixed methods evaluation including a quantitative outcome evaluation to assess tentative effectiveness of the training and a qualitative and quantitative process evaluation to derive indications for future implementation in different clinical settings.

For outcome evaluation, the selected outcomes cover all four levels of the Kirkpatrick model: reaction, learning, behavior and results [21]. Measurements will be performed at baseline (t0), directly after the training (t1) and 4 weeks after the training (t2). Basic demographic information (e.g., age, sex, years of work experience, previous participation in CSTs), will be assessed from all study participants at baseline. Table 1 provides an overview of the applied measures including the time points for their assessment. All measures were selected according to a detailed review of existing literature. Additionally, recommendations of the study advisory board of international experts specialized in healthcare communication were taken into account. Outcome measures, which have to be translated and/or adapted for this study, will be pre-tested via cognitive interviews with 5 to 8 nurses to assess their comprehensibility [31].

**Table 1:** Overview on measures using a modified version of Kirkpatrick’s four levels of training evaluation [21]

		t0	t1	t2
**Level 1: Reaction** (What are participants’ reactions to the CST?)				
Rating of the training (self-developed)			X	
**Level 2: Learning** (What is the impact of the CST on participants’ self-efficacy regarding communication with patients, knowledge about patient-centered communication, communication skills, and attitudes towards medical communication?				
**Primary outcome**: Self-efficacy regarding communication skills with patients (SE-12-G)		X	X	X
Subjective knowledge (self-developed)		X	X	X
Objective knowledge (self-developed)		X	X	X
Attitudes towards medical communication (Attitudes Towards Medical Communication Scale)		X		X
**Level 3: Behavior** (What is the impact of the CST on participants’ communication behavior with patients?)				
Communication skills from external observers’ perspective (SPAs to assess communication skills)				X
Communication skills from simulation patients’ perspective (EPAT items (short version))				X
Self-assessed communication skills applied at SPA (EPAT items (short version), adapted to HCPs)				X
Covariate: Performance test anxiety (PTA)				X
**Level 4: Results** (What is the impact of the CST on participants’ work-related mental burden?)				
Professional fulfillment (PFI-G)		X	X	X
Depression & anxiety (PHQ-4)		X	X	X
Subjective working capacities (dimension 1 and 2 of WAI)		X	X	X
**Covariate** (What factors may have an influence on the results of the four levels?)				
Impact of contamination (self-developed)	IG	X		
	CG	X	X	X
Perceived person-centeredness of healthcare settings (EPAT, short version)		X		X

*CG* waitlist-control group, *CST* communication skills training, *EPAT* Experienced Patient-Centeredness Questionnaire, *IG* intervention group, *PFI-G* German version of Professional Fulfillment Index, *PHQ-4* Patient Health Questionnaire 4, *PTA* Performance Test Anxiety, *SE-12-G* German version of Self-Efficacy of Communication Skills Scale, *SPA* standardized patient assessment, *WAI* Work Ability Index

#### Primary outcome

As the primary outcome, we chose self-efficacy regarding nurses’ communicating with patients. According to Albert Bandura (1997) self-efficacy is the belief in one's own ability to successfully deal with certain tasks or situations [32]. *S*elf-efficacy has been shown to be positively related to HCPs’ performance in communication [33]. Thus, the concept of self-efficacy has been frequently used to assess the impact of CST on HPs [11, 33, 34]. Self-efficacy regarding communication skills with patients will be assessed using the German version of the 12-item *Self-Efficacy of Communication Skills scale (SE-12).* The original scale was developed by Axboe et al. [35] and has already been used as primary outcome in other studies to evaluate CSTs for nurses [11]. Items on this one-dimensional scale can be answered on a scale from 1 (“very uncertain”) to 10 (“very certain”) with an additional option to rate items as “not relevant”. In a previous study by the research team, the SE-12 was translated and adapted into German following the team approach consisting of translation, review, adjudication, pretesting and documentation (TRAPD) [36] and psychometrically tested by Frerichs et al. [37]. Self-efficacy will be assessed at all measurement points (t0, t1, t2), with t1 being the primary endpoint.

#### Secondary outcomes

##### Rating of CST

Participants will be asked to rate the CST regarding its content, used materials and didactic style. Therefore, a short self-developed measure will be adapted from previous studies on CST evaluation to fit trainings’ content. The rating of the CST will be assessed directly after the training (t1).

##### Subjective knowledge

Subjective knowledge of the participants about patient-centered communication will be assessed via self-developed items adapted from a previous study [38] and based on the content of the four training modules. It will be assessed at all measurement points (t0, t1, t2).

##### Objective knowledge

Objective knowledge of participants about patient-centered communication will be assessed via several self-developed multiple-choice questions, based on the trainings’ content. It will also be assessed at all measurement points (t0, t1, t2).

##### Attitudes towards medical communication

Attitudes of participants towards medical communication will be measured using the 12-item *Attitudes Towards Medical Communication Scale* (ATMCS) [39]. Items on this one-dimensional scale can be answered on a Likert scale from 1 (“strongly disagree”) to 5 (“strongly agree”). The Spanish version of this scale was adapted and psychometrically evaluated in a nursing sample [40]. A German version of the ATMCS will be translated using the TRAPD translation protocol [36], and adapted to our target group. The German version of the *Attitudes Towards Medical Communication Scale* will be assessed at baseline (t0) and four weeks after the training (t2).

##### Communication skills from external observers’ perspective

Participants’ communication skills from an external observers’ perspective will be assessed via *Standardized Patient Assessments* (SPAs) [41]. Experienced researcher from other research groups within the UKE, who are not involved in this study, will be trained by the research team and will then carry out the rating. They will neither know participants’ name or their department/ward nor if participants are member of the IC or WG. The SPA scenario describes a well-known situation in nurses' daily work and was developed and tested together with nursing professionals. The rating tool for the SPA contains various general communication skills like introducing oneself to the patient, active listening or question asking. Additionally, as such a SPA situation for nursing professionals has not been described in prior research, we planned the SPA assessment as an explorative evaluation. Participants’ communication skills will be assessed four weeks after the training (t2).

To assess the level of anxiety associated with performing an SPA, the *Performance Test Anxiety questionnaire* (PTA) [42] will be applied. The PTA was derived by taking two factors from the Three-Factor Anxiety Inventory (TFAI) [43], a tool that measures performance anxiety. Factor 1 of the PTA consists of the subscale ‘cognitive anxiety’ and assesses worry and self-focus attention. Factor 2 consists of the subscale ‘physiological anxiety’ and assesses autonomic hyperactivity and somatic tension. Items can be answered on a 5-point-Likert scale from 1 (“totally disagree”) to 5 (“totally agree”). A German version of the PTA will be translated using the TRAPD translation protocol [36]. The PTA will also be assessed four weeks after the training (t2), directly before participants take part in the SPAs.

##### Communication skills from simulation patients’ perspective

Participants’ communication skills during an SPA will be assessed by the simulated patient using several items of the short version of the German measure *Experienced Patient-Centeredness Questionnaire* (EPAT) [44]. This measure assesses experienced patient-centeredness and has recently been developed and psychometrically tested by the research team. Items can be answered on a 6-point-Likert scale from 1 (“completely disagree”) to 6 (“completely agree”). It will be assessed four weeks after the training directly after performing the SPA (t2).

##### Self-assessed communication skills

Participating nurses will assess their own communication skills during SPAs by rating several items of the EPAT [44], which will be adapted to HCPs by the study team. This measure will also be assessed four weeks after the training directly after performing the SPA (t2).

##### Professional fulfillment

Nurses’ professional fulfillment and overall burnout will be assessed using the 16-item *Professional Fulfillment Index* (PFI) [45]. This three-dimensional measure comprises three main scales (‘HCP’s professional fulfillment’, ‘work exhaustion’ and ‘interpersonal disengagement’) as well as an overall burnout score. Items can be answered on a scale from 0 (“not at all”) to 4 (“extremely”). A German version of the PFI was recently translated and adapted by the research team. The PFI will be assessed at all measurement points (t0, t1, t2).

##### Depression and anxiety

To assess symptoms of depression and anxiety, the German version of the *Patient Health Questionnaire 4* (PHQ-4) [46] will be applied. The PHQ-4 is a two-dimensional scale with four items, which can be rated on a scale from 0 (“not at all”) to 3 (“nearly every day”). The PHQ-4 will be assessed at all measurement points (t0, t1, t2).

##### Subjective working capacities

Participants’ subjective working capacities will be assessed via two dimensions of the German version of the *Work Ability Index* (WAI) [47]. Dimension one (‘subjective estimation of current work ability compared with lifetime best’) comprises one item and can be answered on a scale from 1 (“totally unable to work”) to 10 (“best working capacity”). Dimension two (‘subjective work ability in relation to job demands’) comprises two items and can be answered on a scale from 1 (“very bad”) to 5 (“very good”). The WAI will be assessed at all measurement points (t0, t1, t2).

##### Impact of contamination

To measure the impact of contamination, a self-developed item will assess the extent to which participants from CG have received information about the trainings’ content from colleagues of the IG. This item will be assessed in the CG at all measurement points (t0, t1, t2) and in the IG only at baseline (t0).

##### Perceived patient-centeredness of healthcare settings

Perceived patient-centered quality of the care environment within the wards, participants are working in, will be assessed using the short version of EPAT [44], which was adapted for HCP. This measure will be assessed prior to the training (t0) and four weeks after the training (t2).

#### Process evaluation

The purpose of the process evaluation is to analyze deviations from the study protocol as well as factors which influence the roll-out of the CST (e.g. barriers and facilitators). Based on results of the process evaluation, indications for a future implementation of the CST should be derived. We will use the CFIR [19] as well as implementation outcomes defined by Proctor et al. [48] to guide the process evaluation.

For process evaluation, qualitative and quantitative data will be collected during the entire course of the RCT. Qualitative data will include [1] field notes of the research team regarding observations of interactions between members of the research team and clinical stakeholders, participant recruitment and data collection processes, [2] minutes of research team meetings regarding data collection during process evaluation, and [3] short interviews with clinical stakeholders who participated and who did not participate in the CST, applying a purposive sampling strategy with maximum variation approach. For those, clinical stakeholders of different professions and different hierarchy levels will be included and interviews will be audio-recorded. Those data will gain insights in e.g. acceptability, appropriateness and feasibility of the CST. Quantitative data will include quantitative key indicators on dropout rates, training participation, duration, and numbers of trainings per department. Those data will gain insights into reach, dose and fidelity of the CST and could be used to adapt the process of CST roll-out to promote participant retention throughout the whole study if necessary.

### Data monitoring

The process of data collection and management will be documented by the research team continuously during all measurement points. In regular meetings, the process will be discussed and adjustments will be made if necessary. All data will be stored at a secure server of the department of Medical Psychology. Only members of the research team have access to the secure server. To ensure data protection throughout the trial, a data protection protocol was conducted. Data deletion is carried out using a data-cleaning protocol.

### Data analysis

Prior to analysis of quantitative data, a missing data analysis will be conducted and cut-off criteria for including instruments in analysis will be applied. Multiple imputation will be used to handle remaining missing data. Descriptive outcome analysis and process evaluation will be performed by calculating frequencies and percentages for categorical data, medians and interquartile ranges for ordinal or non-normally distributed quantitative data, and means and standard deviations for approximately normally distributed data. Quantitative outcomes will be analyzed by linear mixed models including treatment group, time, and their interaction as fixed effects. Hierarchical data structures (e.g., clustering within departments) will be accounted for by letting the intercept vary randomly across clusters. Categorical and ordinal outcomes will be analyzed by corresponding models which use an appropriate link (e.g., logistic for binary variables). We will report 95 percent confidence intervals for all estimated parameters. The level of statistical significance for the primary confirmatory analysis will be set at p<.05. All other analyses will be considered exploratory. Qualitative data of the process evaluation will be analyzed according to principles of content analysis [49]. Quantitative data of outcome and process evaluation will be entered into and analyzed with SPSS (IBM SPSS Statistics, V.23) by members of the research team consecutively. To ensure high data quality, 20% of the quantitative data will be entered double for quality control. Qualitative data of process evaluation will be imported into and analyzed with MAXQDA software (VERBI GmbH, Berlin, Germany).

### Validity and reliability / rigour

This study uses a rigorous study design, i.e., a RCT with a CG and stratified randomization. For outcome evaluation, a range of measures with high validity and reliability will be used. One measure, the *Attitudes Towards Medical Communication Scale* [39],will be translated into German using the TRAPD translation protocol [36] and psychometrically tested in parallel to the RCT. Additionally, we will conduct cognitive interviews for all translated and adapted measures to ensure comprehensibility by the target group. Furthermore, the measures as well as the newly developed CST will be pre-tested in a sample, which will be comparable to the target group. Single items as well as the SPAs will be developed by the research team according to the trainings’ content and pre-tested. Blinded double data entry of quantitative data will be performed. The study team was supported by an independent advisory board of international experts specialized in healthcare communication. For preparing this study protocol, we followed the Standard Protocol Items: Recommendations for Interventional Trials 2013 (SPIRIT 2013) [50].

## Discussion

This study closes the research gap of a needs-based developed and systematically evaluated CSTs for nursing professionals in Germany. Since this patient-centered CST was developed according to previously analyzed needs of nurses, it will directly address communicative challenges of their daily work routine. We expect to improve self-efficacy regarding communication, communication skills and mental burden of participating nurses. Furthermore, we will derive indications for future implementation of the CST into routine clinical practice. In case of a proven effectiveness of the CST for nurses, an implementation in diverse clinical settings (including e.g. geriatric or rehabilitation facilities) should be conducted and evaluated in future implementation studies. Thereby, additional and more distant implementation outcomes, especially the impact on patient-related outcomes (e.g. satisfaction with communication with HCPs, anxiety) could be addressed as they are not part of the study at hand. Finally, our results may be useful for other health care institutions and implementation researchers when planning or implementing CST for nurses.

### Dissemination

Results of this study will be presented at national and international conferences. Furthermore, results will be published in relevant journals (peer reviewed, open access) to ensure accessibility for clinicians, researchers and other stakeholders, including patients. Press releases, in-house newsletters and social media will also be used for dissemination. In addition, a project website provides information about study progress (UKE - Institut und Poliklinik für Medizinische Psychologie - KOMPAT Projekt).

### Limitations

There will be several limitations of this study. First, it is a single-center study conducted at one academic medical center in Germany. Thus, generalizability to other healthcare settings and/or other countries is limited and should be analyzed in future studies. Second, our sampling approach might lead to a self-selection bias of participants, who are specifically interested in the topic. To improve diversity of the sample, we will ensure to include participants of diverse clinical backgrounds and work experience. We plan to apply stratified randomization, where participants will be randomized within strata (i.e., departments of the UKE). Thus, there will be a chance of contamination within these groups as participants of the IG and CG might work closely together within a ward. To reduce contamination processes to a minimum, participants of both groups will be informed about disadvantages of contamination for the study’s results and will be asked not to share content of the training or the evaluation with other colleagues. Additionally, the evaluation questionnaire will contain one item on contamination to include this factor as a covariate in the statistical outcome evaluation if necessary.

## Conclusion

This is the first study systematically evaluating a needs-based patient-centered CST for nursing professionals in Germany. Results will give insights into effects of the training on self-efficacy, communication behavior and mental burden as well as other relevant outcomes. A thorough process evaluation will help to derive initial indications for future implementation of the training.

## Data Availability

Not applicable.

## Abbreviations

ATMCS: Attitude towards medical communication scale
C-CG: Calgary-Cambridge Guide
CFIR: Consolidated Framework for Implementation Research
CG: Waitlist-control group
CST: Communication skills training
EPAT: Experienced Patient-Centeredness Questionnaire
HCP: Healtcare professional
IG: Intervention group
PFI: Professional Fulfillment Index
PHQ-4: Patient Health Questionnaire-4
PTA: Performance Test Anxiety Questionnaire
RCT: Randomized Controlled Trail
SE-12: Self-Efficacy of Communication Skills Scale
SPA: Standardizedd Patient Assessment
SPIRIT: Standard Protocol Items: Recommendations for Interventional Trials
TFAI: Three-Factor Anxiety Inventory
TRAPD: Translation, Review, Adjudication, Prestest, Documentation
UKE: University Medical Center Hamburg-Eppendorf
WAI: Work Ability Index
